# Adherence to the Japanese Physical Activity Guideline During Early Childhood Among Rural Preschoolers: A Cross-sectional Study

**DOI:** 10.2188/jea.JE20190320

**Published:** 2021-03-05

**Authors:** Noritoshi Fukushima, Takafumi Abe, Jun Kitayuguchi, Chiaki Tanaka, Shiho Amagasa, Hiroyuki Kikuchi, Shinpei Okada, Shigeho Tanaka, Shigeru Inoue

**Affiliations:** 1 Department of Preventive Medicine and Public Health, Tokyo Medical University, Tokyo, Japan; 2 Center for Community-Based Healthcare Research and Education (CoHRE), Shimane University, Shimane, Japan; 3 Physical Education and Medicine Research Center UNNAN, Shimane, Japan; 4 College of Health and Welfare, J. F. Oberlin University, Tokyo, Japan; 5 Physical Education and Medicine Research Foundation, Nagano, Japan; 6 Department of Nutrition and Metabolism, National Institute of Health and Nutrition, National Institutes of Biomedical Innovation, Health and Nutrition, Tokyo, Japan

**Keywords:** guideline, physical activity, preschoolers, Japanese

## Abstract

**Background:**

Physical activity (PA) guidelines for early childhood have been established worldwide, and adherence to PA guidelines has been utilized to assess the effectiveness of policies regarding PA promotion. Although there is a Japanese PA guideline for preschoolers, little is known about adherence to this recommendation. This study examined and compared proportions of meeting the Japanese PA guideline among preschoolers.

**Methods:**

Participants comprised 821 children aged 3–6 years from all 21 preschools and childcare facilities (hereafter collectively “preschools”) within Unnan City, Shimane Prefecture, Japan. Data on PA levels were collected through a parent-report questionnaire in accordance with the Japanese PA guideline. This guideline recommends that preschoolers perform PA for at least 60 minutes every day. Analyses included descriptive statistics, chi-squared, and Mann–Whitney’s tests to compare adherence to the PA guideline.

**Results:**

Data of 441 participants from 20 preschools were analyzed. Of these, 292 (66.2%) preschoolers met the PA guideline. Boys (70.2%) showed a significantly higher proportion of meeting the PA guideline than girls (61.2%; *P* = 0.048). Proportions of meeting the PA guideline among preschool grades were not statistically different. Prevalence rates of meeting the PA guideline among 20 preschools considerably varied from 14.3% to 100% (*P* = 0.007).

**Conclusions:**

Two-thirds of preschoolers met the Japanese PA guideline, while adherence to PA recommendations differed between genders. Moreover, there were distinct variations of adherence to PA guideline among preschools. Possible determinants that cause the differences in adherence to the PA guideline at the individual and preschool-levels should be further evaluated.

## INTRODUCTION

The preschool-aged period (mostly encompassing 3–5 years of age) plays an important role to establish fundamental physical activity (PA) levels, and acquired PA habits would track from early childhood into their later life.^1^ Higher levels of PA in preschool-aged children are associated with several physical and psychosocial health benefits, such as obesity prevention, bone and skeletal health, motor skill development, reduced cardiovascular disease risks, and increased cognitive development, academic achievement, and prosocial behavior.^2^^–^^5^ Moreover, preschoolers’ adherence to PA guidelines provides useful information that helps policy makers understand their PA levels. Since there are different PA guidelines worldwide, each country has separately investigated its proportions of children during early childhood who adhered to its own PA guidelines.^6^^–^^9^

In Japan, the PA guideline for preschoolers aged 3–6 years, a document called “Youjiki Undo Shishin,” has been released by the Ministry of Education, Culture, Sports, Science and Technology (MEXT) in 2012.^10^ In the Japanese PA guideline, the MEXT provides question items to measure PA levels among preschoolers. It has been suggested that a self-reported PA questionnaire would be a useful survey instrument for undertaking population surveillance for the purpose of estimating the prevalence of PA such as the percentage of meeting national guidelines.^11^ Nevertheless, there are no available data on adherence to the Japanese PA guideline from the MEXT in early childhood. In addition, little is known about whether there are any differences in adherence to the Japanese PA guidelines among preschools. In Japan, a Nationwide Survey on Families and Children by the Ministry of Health, Labour and Welfare reported that 99.2% of preschoolers attend either preschool or childcare facilities by the age of 5 years^12^; hence, it is important to examine whether variations of adherence to PA guidelines exist among preschools and childcare facilities.

Therefore, the aims of this study were twofold. First, we investigated the proportions of adherence to the Japanese PA guideline of children in early childhood, (Youjiki Undo Shishin) among preschoolers aged 3–6 years. This process was conducted in accordance with the procedures set forth in the Guidebook of the MEXT PA guideline, and the children were stratified by gender, preschool grades, and types of preschools. Second, we compared the proportions of adherence to the Japanese PA guideline across preschools and childcare facilities that participated in this survey.

## METHODS

### Participants and data collection

In total, 821 students from Unnan City, a rural municipality within Shimane Prefecture, Japan, were listed in this study. The participants were recruited from all preschools and childcare facilities within the city: 10 nursery schools (Hoikusho; *n* = 403), 5 kindergartens (Yochien; *n* = 68), and 6 authorized centers for early childhood education and care (ECEC; or Nintei kodomo en; *n* = 350). To briefly explain, Japanese nursery schools provide full-day care to children aged 0–6 years, kindergartens provide education to children aged 3–6 years, and ECECs provide both full-day care and education to children aged 0–6 years (education starts when they are 3 years old). On the first April after they become 6 years old, the children are compulsorily sent to primary school.

Hereinafter, nursery schools, kindergartens, and ECECs will be collectively referred to as “preschools.” Within the city hall, this research was announced to all 21 preschools by the Board of Education and Children Policy division from Unnan City. Each principal from the eligible preschools was entrusted with the decision to cooperate in this study. Principals who decided not to cooperate in this study were asked to report their reason, if possible. Prior to participation, a written informed consent was obtained from each child’s parents. Then, the questionnaires were distributed to parents (who responded on behalf of their children) and collected at each preschool in October 2017. An ethics approval was provided by the Physical Education and Medicine Research Center Unnan.

### Measurements of physical activity among preschoolers

Physical activity was assessed using a questionnaire with the Japanese PA guideline.^10^ Although not formally validated, it was originally developed by members of the committee who established this guideline, and was meant to be a survey tool for PA levels among preschoolers. The assessment of the children’s PA in this study was conducted using the parent-report questionnaire based on the Japanese PA guideline; this method has already been widely used in many national health surveys (eg, England, Scotland, and Australia).^13^^–^^15^ The children’s parents were asked to report the average amount of time that their children had spent in active play. The question was as follows: “How many average hours per day did you play?” (There was a footnote that stated, “including any bodily play and exercise [eg, play tag, play hide-and-seek, ball play, slide play, sand play, etc]), and time spent walking to preschool.” For weekdays, answers were divided into two types of situation and were recorded separately: average minutes per day in preschool, and average minutes per day outside preschool. For weekends, answers were not divided and only provided the average minutes per day. The children’s play times were reported as continuous variables (minutes/day). In a supplementary analysis, weighted overall PA time throughout the week (minutes/day in a week) was calculated using estimation from supplementary analyses; (total PA time [minutes/day] during weekdays × 5 [days] + total PA time [minutes/day] during weekends × 2 [days])/7 (days).

### Definition of meeting the Japanese PA guideline for preschoolers

According to the PA guideline for preschoolers, children who played at least 60 minutes per day on both weekdays and weekends were considered to adhere to the guideline. The PA guideline further instructed that, since weekday answers were divided into two types (ie, in preschool and outside of preschool), weekday answers should be summed up to assess children’s play time.

### Sociodemographic variables

Before distributing the questionnaire to each preschool, their types (eg, nursery school) were recorded. The children’s gender and preschool grades were obtained through the questionnaire. In Japan, preschool grades are divided by age: children aged 3–4 years (ie, all children who were 3 years old at the beginning of this fiscal year and will become 4 years old through the year) belong to the first grade (Nensho class); children aged 4–5 years belong to the second grade (Nenchu class); and children aged 5–6 years belong to the third/final grade (Nencho class).

### Statistical analyses

To describe the characteristics of the sample (gender, preschool grades, and types of preschools) and the proportion of preschoolers who adhered to the PA guideline, frequencies were presented. Continuous variables were presented as means and standard deviations (SDs) or medians and 25^th^–75^th^ percentiles, and categorical variables were reported as percentages. Differences in the proportions of adherence to the PA guideline within each characteristic of the sample were assessed using chi-squared tests. Further analyses stratified by gender and preschool grades were conducted to examine the differences in the proportions of adherence to the PA guideline. Continuous variables between the two groups were compared using Student’s *t*-tests or Mann–Whitney’s U test, as appropriate. We set the statistical significance level at *P* < 0.05 for two-sided tests. All analyses were conducted using the IBM SPSS 24 software (IBM Corp., Armonk, NY, USA).

## RESULTS

**Figure 1.**
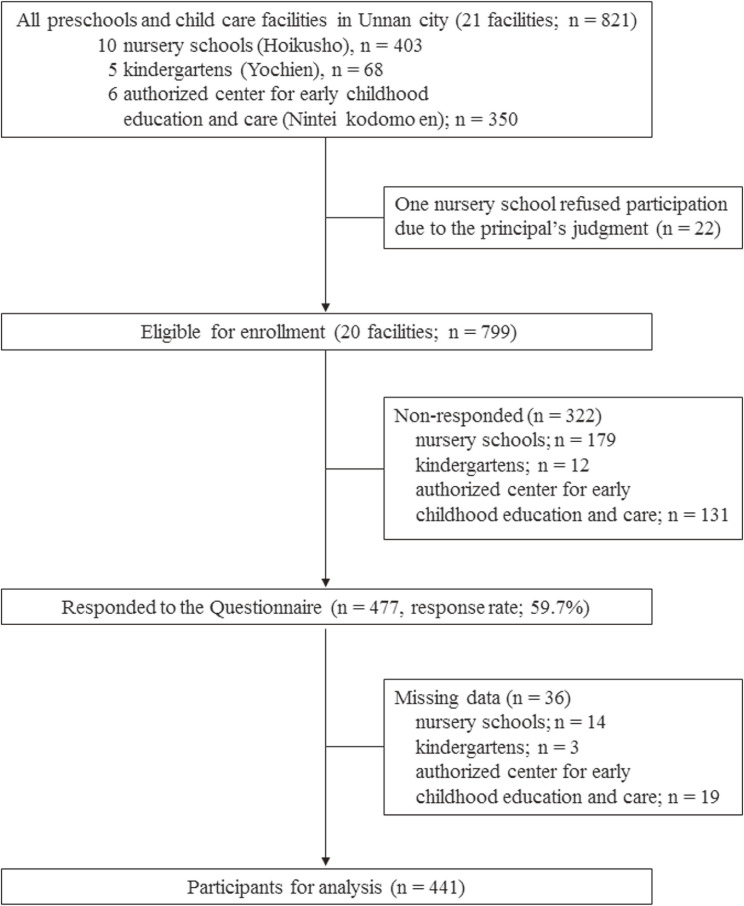
Sampling flow chart

After the study was announced, only one principal (from a nursery school) refused participation in the study without providing a reason. In total, 20 preschools (95.2%) and 799 preschoolers were engaged, with 477 preschoolers’ parents responding to the questionnaire (response rate: 59.7%; Figure 1). There were significant differences in the response rates among the types of preschools (53.1% in nursery schools; 82.4% in kindergartens; and 62.6% in ECEC; *P* = 0.001). Finally, after the exclusion of missing data (*n* = 36), 441 preschoolers (mean age 5.3; SD, 0.8 years; boys: 55.6%) were analyzed (Figure 1). Among our sample, the percentages of preschoolers who attended kindergartens were lower for both boys and girls (13.9 and 9.7%, respectively; *P* = 0.25) as compared to all other types of schools. However, they were not significantly different. The participants’ characteristics are shown in Table 1.

**Table 1.** Participants’ characteristics

	Total (*n* = 441)	Boys (*n* = 245)	Girls (*n* = 196)	*P* value^a^
Preschool grades				
First year	123 (27.9)	69 (28.2)	54 (27.6)	0.17
Second year	153 (34.7)	93 (38.0)	60 (30.6)	
Third/final year	165 (37.4)	83 (33.9)	82 (41.8)	
Types of preschools				
Nursery schools	188 (42.6)	107 (43.7)	81 (41.3)	0.25
Kindergartens	53 (12.0)	34 (13.9)	19 (9.7)	
Authorized center for early childhood education and care	200 (45.4)	104 (42.4)	96 (49.0)	

Number (%).

^a^Comparisons of gender with preschool grades and types of preschools by chi-squared tests.

**Figure 2.**
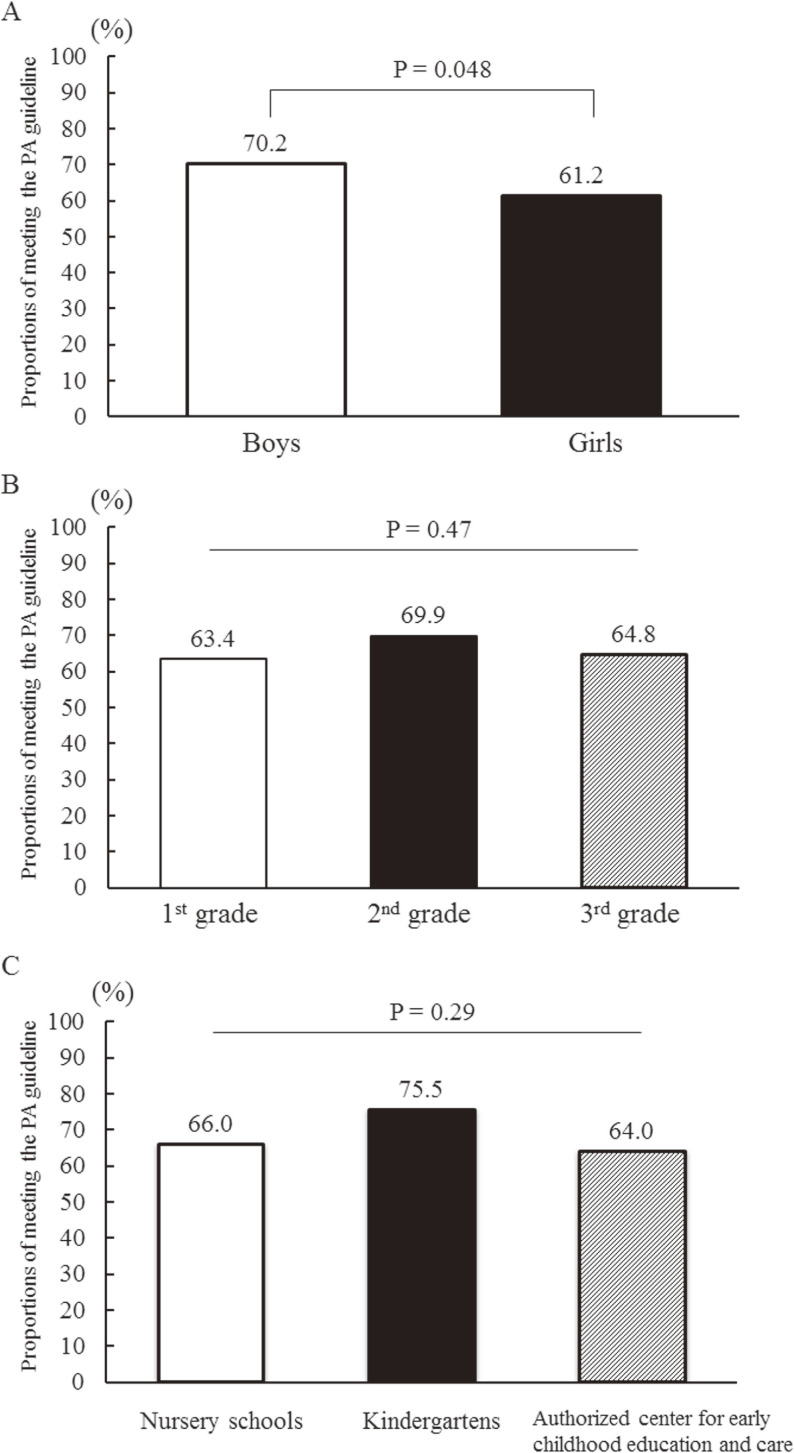
Participants’ adherence to the Japanese physical activity (PA) guidelines. A) Comparisons between boys and girls, B) comparisons among school grades, and C) comparisons among different types of preschools.

Of the 441 participants, 292 (66.2%) preschoolers adhered to the PA guideline for children in early childhood. Boys had higher proportions of adherence to the PA guideline as compared to girls (70.2% for boys; 61.2% for girls; *P* = 0.048; Figure 2A). There were no significant proportion differences in adherence to the PA guideline among preschool grades (63.4% for the first year; 69.9% for the second year; and 64.8% for the third/final year; *P* = 0.47; Figure 2B), and among types of preschools (66.0% for nursery school; 75.5% for kindergartens; and 64.0% for ECEC; *P* = 0.29; Figure 2C).

**Figure 3.**
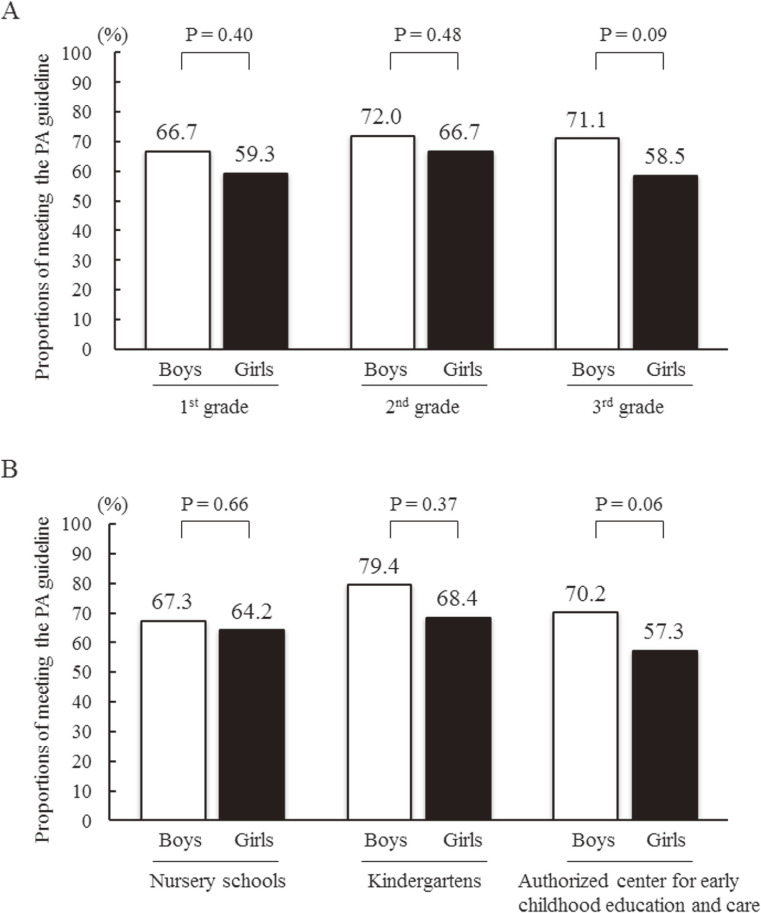
Comparisons of the participants’ proportions of adherence to the Japanese physical activity (PA) guidelines between genders, A) stratified by school grades, and B) stratified by types of preschools.

After stratifying data by preschool grades and types of preschools, boys tended to have higher proportions of adherence to the PA guideline in the third grade as compared to girls (*P* = 0.09; Figure 3A) and in ECEC (*P* = 0.06; Figure 3B). After re-stratifying data in Figure 3A by gender, there were no significant differences among preschool grades in proportions of adherence to the PA guideline (*P* = 0.74 for boys, *P* = 0.58 for girls). After re-stratifying data in Figure 3B by gender, there were no significant differences among types of preschools in proportion of adherence to the PA guideline (*P* = 0.40 for boys, *P* = 0.51 for girls, respectively).

**Figure 4.**
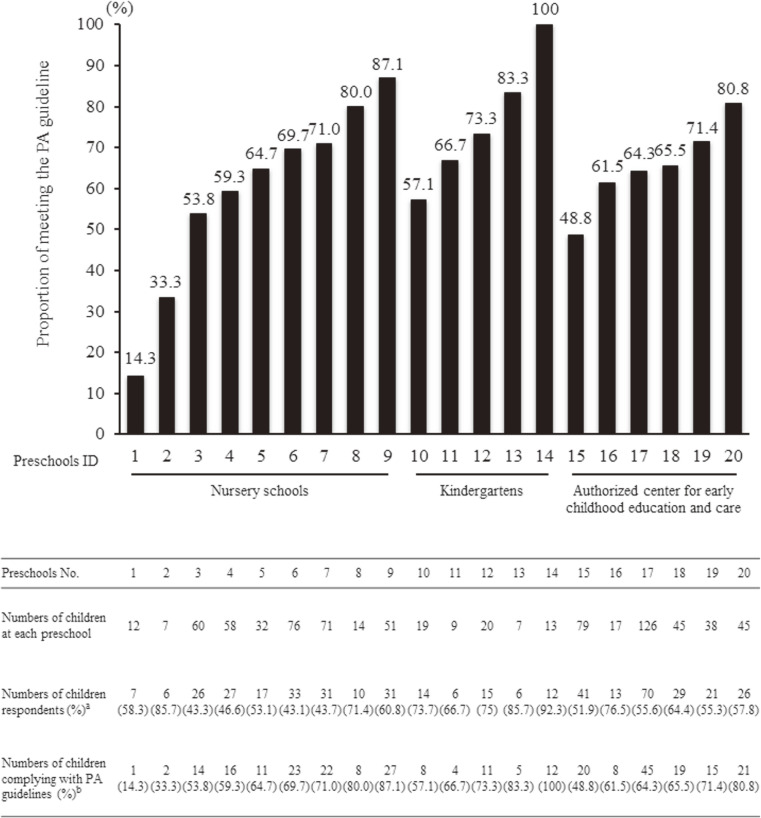
Descriptive variations of the proportions of adherence to the Japanese physical activity (PA) guidelines among the 20 preschools; numbers for children registered at each preschool; number and proportions of respondents to the questionnaire at each preschool; and number and proportions of children who adhered to the PA guidelines among respondents to the questionnaire at each preschool.  ^a^Proportion (%) as response rate at each preschool.  ^b^Proportion (%) of those meeting the PA guideline at each preschool.

The variations of adherence to the PA guideline among preschools are shown in Figure 4. The distributions of prevalence rates of adherence to the PA guideline among the 20 preschools considerably varied, ranging from 14.3% to 100% (*P* = 0.007; Figure 4).

In terms of time spent engaging in PA during weekdays and weekends separately, 75 children reported spending ≥60 minutes/day engaging in PA only on weekdays, and 42 children reported spending ≥60 minutes/day engaging in PA only on weekends (Table 2). Descriptive play times reported by parents are shown as continuous values in Table 3. The amount of total time spent engaging in PA during weekdays (ie, that inside as well as outside of preschool) was higher as compared to that during weekends, irrespective of gender, preschool grade, and type of preschool. On weekdays, the amount of PA time during preschool was significantly higher than that out of preschool, regardless of gender, preschool grade, or type of preschool. The weighted overall PA time throughout a week is provided as supplementary information. When considering this supplementary calculated PA time, the proportions of the corresponding prevalence rates for ≥60 minutes of daily PA changed to 79.7% (81.6% for boys and 77.3% for girls), and there was no significant differences with regard to gender (*P* = 0.26).

**Table 2.** Participants’ numbers on the amount of time they spent in physical activities on weekdays and weekends

PA time on weekdays	PA time on weekends		Total number

	<60 minutes/day	≥60 minutes/day	
<60 minutes/day	32 (7.3)	42 (9.5)	74 (16.8)
≥60 minutes/day	75 (17.0)	292 (66.2)	367 (83.2)

Total number	107 (24.3)	334 (75.7)	441 (100)

PA, physical activity.

Number (%).

**Table 3.** Participants’ levels of physical activity overall, on weekdays, and on weekends by gender, preschool grade, and type of preschool

	Weighted overall PA time^a^ (minutes/day in a week)		Weekdays (minutes/day)						Weekends (minutes/day)	
	
			Total PA time^b,c^		PA time in preschools		PA time out of preschools		Total PA time^b,c^	
		
	mean (SD)	median (25, 75 percentile)	mean (SD)	median (25, 75 percentile)^c^	mean (SD)	median (25, 75 percentile)^d^	mean (SD)	median (25, 75 percentile)^d^	mean (SD)	median (25, 75 percentile)^c^
Overall	145 (107)	117 (64, 197)	164 (128)	120 (60, 240)	151 (105)	120 (60, 120)	36 (30)	30 (10, 60)	97 (88)	60 (60, 120)
Sex										
Boys	153 (109)	124 (67, 206)	172 (128)	140 (70, 240)^e^	158 (108)	120 (60, 240)^e^	38 (45)	30 (10, 60)^e^	106 (94)	60 (60, 120)^d^
Girls	135 (105)	103 (60, 179)	153 (128)	120 (60, 210)^e^	143 (101)	120 (60, 180)^e^	33 (44)	30 (10, 40)^e^	84 (78)	60 (30, 120)^d^
School grade										
First year	132 (93)	103 (65, 177)	149 (115)	120 (60, 210)^e^	141 (100)	120 (60, 180)^e^	30 (31)	30 (0, 32)^e^	87 (74)	60 (40, 120)^d^
Second year	152 (116)	120 (67, 206)	170 (136)	130 (67.5, 240)^e^	156 (109)	120 (60, 240)^e^	40 (56)	30 (10, 60)^e^	105 (98)	60 (60, 120)^d^
Third year	133 (96)	120 (60, 206)	170 (130)	130 (60, 240)^e^	155 (106)	120 (60, 240)^e^	36 (40)	30 (10, 60)^e^	96 (87)	60 (55, 120)^d^
Type of preschool										
Nursery schools	138 (101)	103 (60, 197)	154 (120)	120 (60, 235)^e^	153 (105)	120 (60, 240)^e^	25 (29)	20 (0, 30)^e^	94 (87)	120 (60, 230)^d^
Kindergartens	154 (89)	144 (81, 206)	173 (107)	150 (85, 240)^e^	141 (92)	120 (60, 180)^e^	47 (26)	60 (30, 60)^e^	97 (72)	60 (60, 120)^d^
ECEC	150 (117)	118 (62, 193)	171 (140)	130 (60, 240)^e^	153 (109)	120 (60, 240)^e^	43 (56)	30 (10, 60)^e^	99 (93)	60 (42.5, 120)^d^

ECEC, authorized center for early childhood education and care; PA, physical activity; SD, standard deviation.

^a^Weighted overall PA times throughout a week (minutes/day in a week) were calculated by the following estimation in the supplementary analyses: (total PA time [minutes/day] during weekdays × 5 [days] + total PA time [minutes/day] during weekends × 2 [days])/7 (days).

^b^Total PA times during weekdays includes PA time inside and outside of preschool.

^c^Comparisons between total PA time on weekdays and that on weekends by Mann–Whitney tests.

^d^Comparisons between PA time during inside of preschool and that outside of preschool during weekdays by Mann–Whitney tests.

^e^Statistically significant (*P* < 0.001) according to Mann–Whitney tests.

## DISCUSSION

The main results of this study indicated that 1) two-thirds of the participants (66.2%) adhered to the Japanese PA guideline, named Youjiki Undo Shishin; 2) boys had higher proportions of adherence to the PA guideline as compared to girls; 3) and adherence to the PA guideline considerably varied among the 20 participant preschools. To the best of our knowledge, this is the first study that has reported the proportions of adherence to the Japanese PA guideline for children in early childhood following the procedures set forth by the MEXT.

Our results showed that 66.2% of the preschoolers adhered to the Japanese PA guideline for children in early childhood. The proportions of adherence to the PA guideline in this study were higher in comparison to those of other countries, such as 28% in England (children aged 5–7 years),^13^ 45% in Scotland (children aged 5–7 years),^14^ and 36% in Australia (children aged 5–8 years).^15^ The average times spent performing PA in a usual week were examined using the Japanese PA guideline,^10^ while most of the aforementioned countries asked the amount of time in the past 7 days prior to the survey and evaluate whether preschoolers spent enough PA times every day.^13^^–^^15^ In this regard, the evaluations of adherence to the Scottish PA guidelines changed since 2017: it went from asking average PA times during the week to asking PA times for each day of the week (ie, Monday–Sunday). After this modification, a notable decline in adherence to their PA guidelines was observed (33% of children aged 5–15 years in the Scottish Health Survey from 2017 as compared with 73% of children aged 2–15 years in the Scottish health survey from 2015).^14^^,^^16^ Our findings on relatively higher proportion of adherence, compared to other countries, might be affected by the different methodological measurements used to quantify PA times based on either estimating average PA time or estimating daily PA time.

In addition, our results showed that boys had higher levels of adherence to the PA guideline as compared to girls. A systematic review on the topic showed that several studies concurred with this finding, and reported that boys were more active than girls.^17^ We previously reported that the prevalence of meeting the PA guideline for children and adolescents in girls was lower as compared to that in boys among Japanese children aged 9–15 years.^18^ Our results extend these relevant pieces of evidence from Japanese children to preschoolers, showing that the gender differences were already observed in the preschool period. Regarding gender differences in PA levels among preschoolers, a study reported that preschool girls preferred playing inside/drawing/doing craft than being physically active,^19^ which was in part associated with their lower PA levels. Moreover, PA levels among children in early childhood depend on parenting styles, such as encouraging or restricting their children’s PA.^20^^,^^21^ In addition, parenting styles may differ according to the child’s gender, such as the circumstance that boys tend to be allowed to play outdoors alone as compared to girls.^22^ In this study, proportions of adherence to the PA guideline tended to be different between boys and girls in the third year of preschool and ECEC. Thus, socio-ecological approaches that consider preferences, gender, and different parenting styles, potentially including age and types of preschools, could potentially be a well-suited strategy model when developing interventions targeted at promoting PA in early childhood, especially for preschool girls.^23^

In this study, the reported PA levels on weekdays were higher than those on weekends among all categories. However, there were some inconsistent results obtained from comparisons between weekday and weekend PA levels.^24^^,^^25^ It may be more difficult for parents to report how much time their children spend playing at preschools on weekdays because they could not directly observe their children’s PA levels. In addition, parents are naturally prone to perceive their children as active.^26^ Basterfield et al^27^ reported that when parents reported their children’s time spent performing MVPA, they tended to overestimate the levels by around 2 hours per day as compared to reports on studies using accelerometers. These findings suggest that our results may be overestimated, especially PA time during preschool on weekdays.

Although adherence to the PA guideline was not statistically significant between types of preschools, the adherence in kindergarten was the highest among all types of preschool in this study sample. The total response rates remained at a maximum of 59.7%, and the response rates differed significantly among types of preschools, which may affect our results and cause us to underestimate the difference of adherence among types of preschools due to a reduction in statistical power. As mentioned above, in this study, kindergarten had the highest adherence rates among all types of preschools. The children who went to kindergartens usually have shorter times spent at school as compared to that in nursery schools and ECECs, since the latter provide full-day care. Hence, the parents whose children go to kindergarten potentially spend more time with their children, which might make it easier for them to respond to the questionnaire compared to parents of children in nursery schools and ECECs. In addition, parental socioeconomic status, such as a mother’s employment status and the parents’ educational attainment, might be different among types of preschools, which has been reported to affect their children’s PA.^28^ For instance, children whose mothers were engaged in full-time work tended to be less active as compared to those whose mothers were engaged in full-time home duties.^19^ Potential hypothesis could be drawn that Japanese children in kindergartens are potentially more active than those in nursery schools and ECECs because the mothers whose children are in enrolled in the latter tended to work full-time as compared to mothers whose children are still in kindergarten. Further studies are warranted to confirm whether parental status is indeed different among types of preschools and consequently how such status affects children’s PA; a larger sample size would be needed to confirm the relevance of such findings.

It is noteworthy that there were considerable variations in the proportions of adherence to the PA guideline among the different preschools. This may have occurred because the educational ideals related to children’s PA values could have been different among teachers/caregivers at each of the participant preschools. Therefore, each preschool likely had different levels of active/inactive play during preschools and different feedbacks on children’s daily PA levels to their parents. Preschools were one of the ideal settings to promote PA because children spend considerable amounts of their daily time in preschools. These findings suggest that interventions related to PA levels are needed for teachers/caregivers as well as parents. Previous studies have shown that, although it is challenging to promote PA in childcare,^29^ teacher training may be a feasible and effective intervention to promote children’s PA, and could be incorporated into existing curriculum at preschools.^30^ For instance, adopting physically active games that children can play indoors or outdoors, such as dancing, may be considered as potential strategies to improve PA levels for children in early childhood. Previous research on this topic has shown that constant implementation of PA programs by teachers might be a difficult task to accomplish.^31^ However, having a written policy that can facilitate children’s PA opportunities during care time at each preschool could prove to be a supportive strategy not only for increasing children’s PA but also providing behavioral changes for teachers and caregivers related to PA promotion.^32^^,^^33^ In addition, interventions targeted at modifying parenting styles that limit active play are important in increasing PA levels among preschoolers.

Several strengths of this study should also be noted. First, we used the standardized questionnaire presented in the MEXT PA guideline (Youjiki Undo Shishin), which enabled comparison of children’s adherence in different areas across Japan. Accordingly, our results can and should be used as reference data for future research on this topic. Second, sampling was performed as a complete enumeration, and 20 out of 21 preschools from Unnan City participated in this research. Hence, this study contributed to the understanding of the proportions of adherence to the MEXT PA guideline among rural preschoolers.

There are certain limitations to this study. First, the study setting comprised a relatively rural municipality, which hampers generalization because its environmental factors would be different as compared to that in urban areas. Second, the questionnaire for the Japanese PA guideline has not been fully validated; hence, the average PA time measured by this questionnaire is a potentially rough estimate depending on parents’ perceptions. Third, we did not assess how extensively teachers/caregivers share information related to children’s PA with each other, which might affect the parents’ perceptions of their children’s PA levels. Finally, it remains unclear whether preschoolers who adhere to the PA guideline improve their physical and psychosocial health outcomes.

### Conclusions

This study found that 66% of the preschoolers aged 3–6 years in a rural area adhered to the Japanese PA guideline for children in early childhood. Adherence to PA recommendations was different between genders, and boys were more active than girls. Moreover, there were distinct variations of adherence to PA guidelines among preschools. These findings suggested that further studies are needed to seek determinants that cause differences in adherence to the PA guideline at individual and preschool levels. In addition, the association between adherence to the PA guideline and health outcomes among preschoolers, and regional differences (eg, comparisons between urban and rural area) regarding the proportions of adherence to the Japanese PA guideline, should be further evaluated.
